# Supplementary material for: Hybrid Models and Biological Model Reduction with PyDSTool
Source: PLoS Comput Biol. 2012 Aug 9;8(8):e1002628. doi: 10.1371/journal.pcbi.1002628 (PMC3415397; doi:10.1371/journal.pcbi.1002628)
Supplement: Text S4 — Complete source code for the PyDSTool package (version 0.88.120504). Includes API documentation and help files linking to web pages. This file is identical to the current public release on Sourceforge.net. (ZIP) [file pcbi.1002628.s004.zip › PyDSTool/html/PyDSTool.Generator.ODEsystem'.ODEsystem-class.html]

xml version="1.0" encoding="ascii"?


PyDSTool.Generator.ODEsystem'.ODEsystem


| Home | Trees | Indices | Help | PyDSTool |
| --- | --- | --- | --- | --- |

Package PyDSTool ::
Package Generator ::
Module ODEsystem' ::
Class ODEsystem

[hide private]

[frames] | no frames]

# Class ODEsystem

source code

```
           object --+        
                    |        
baseclasses.Generator --+    
                        |    
       baseclasses.ctsGen --+
                            |
                           ODEsystem
```

Known Subclasses:
:   - Radau\_ODEsystem'.Radau\_ODEsystem
    - , Euler\_ODEsystem'.Euler\_ODEsystem
    - , Vode\_ODEsystem'.Vode\_ODEsystem
    - , Dopri\_ODEsystem'.Dopri\_ODEsystem
    - , ADMC\_ODEsystem'.ADMC\_ODEsystem

---

Abstract class for ODE system solvers.


|  |  |
| --- | --- |
| Instance Methods | [hide private] |

|  |  |
| --- | --- |
| AuxVars(self, t, xdict, pdict=None, asarray=False) | source code |

|  |  |
| --- | --- |
| Jacobian(self, t, xdict, pdict=None, asarray=False) | source code |

|  |  |
| --- | --- |
| JacobianP(self, t, xdict, pdict=None, asarray=False) | source code |

|  |  |
| --- | --- |
| Rhs(self, t, xdict, pdict=None, asarray=False) | source code |

|  |  |
| --- | --- |
| \_\_del\_\_(self) | source code |

|  |  |
| --- | --- |
| \_\_getstate\_\_(self) | source code |

|  |  |
| --- | --- |
| \_\_init\_\_(self, kw)  x.\_\_init\_\_(...) initializes x; see x.\_\_class\_\_.\_\_doc\_\_ for signature | source code |

|  |  |
| --- | --- |
| \_\_setstate\_\_(self, state) | source code |

|  |  |
| --- | --- |
| addMethods(self, usePsyco=False)  Add Python-specific functions to this object's methods, accelerating them with psyco, if it is available. | source code |

|  |  |
| --- | --- |
| checkInitialConditions(self, checkauxvars=False) | source code |

|  |  |
| --- | --- |
| cleanupMemory(self)  Clean up memory usage from past runs of a solver that is interfaced through a dynamic link library. | source code |

|  |  |
| --- | --- |
| compute(self, dirn)  This is an abstract class. | source code |

|  |  |
| --- | --- |
| haveJacobian(self)  Report whether ODE system has an explicit user-specified Jacobian associated with it. | source code |

|  |  |
| --- | --- |
| haveJacobian\_pars(self)  Report whether ODE system has an explicit user-specified Jacobian with respect to pars associated with it. | source code |

|  |  |
| --- | --- |
| haveMass(self)  Report whether ODE system has an explicit user-specified mass matrix associated with it. | source code |

|  |  |
| --- | --- |
| prepDirection(self, dirn)  Common pre-integration tasks go here | source code |

|  |  |
| --- | --- |
| set(self, \*\*kw)  Set ODE system parameters | source code |

|  |  |
| --- | --- |
| validateICs(self) | source code |

**Inherited from `baseclasses.ctsGen`**:
`validateSpec`

**Inherited from `baseclasses.Generator`**:
`__copy__`,
`__deepcopy__`,
`__repr__`,
`__str__`,
`addEvtPars`,
`checkArgs`,
`contains`,
`get`,
`getEventTimes`,
`getEvents`,
`info`,
`query`,
`resetEventTimes`,
`resetEvents`,
`setEventICs`,
`showAuxFnSpec`,
`showAuxSpec`,
`showEventSpec`,
`showSpec`

**Inherited from `baseclasses.Generator`** (private):
`_addEvents`,
`_auxfn_getindex`,
`_auxfn_globalindepvar`,
`_auxfn_heav`,
`_auxfn_if`,
`_auxfn_initcond`,
`_generate_ixmaps`,
`_infostr`,
`_kw_process_algparams`,
`_kw_process_allvars`,
`_kw_process_dispatch`,
`_kw_process_events`,
`_kw_process_fnspecs`,
`_kw_process_ics`,
`_kw_process_ignorespecial`,
`_kw_process_inputs`,
`_kw_process_pars`,
`_kw_process_pdomain`,
`_kw_process_reuseterms`,
`_kw_process_system`,
`_kw_process_target`,
`_kw_process_tdata`,
`_kw_process_tdomain`,
`_kw_process_tstep`,
`_kw_process_ttype`,
`_kw_process_varspecs`,
`_kw_process_vfcodeinserts`,
`_kw_process_xdomain`,
`_kw_process_xtype`,
`_makeBoundsEvents`,
`_register`,
`_set_for_hybrid_DS`

**Inherited from `object`**:
`__delattr__`,
`__getattribute__`,
`__hash__`,
`__new__`,
`__reduce__`,
`__reduce_ex__`,
`__setattr__`


|  |  |
| --- | --- |
| Class Variables | [hide private] |

\_needKeys = `['name', 'varspecs']`

\_optionalKeys = `['globalt0', 'checklevel', 'model', 'abseps', ...`

\_validKeys = `['globalt0', 'xdomain', 'tdata', 'tdomain', 'chec...`

**Inherited from `baseclasses.Generator`** (private):
`_querykeys`


|  |  |
| --- | --- |
| Properties | [hide private] |

**Inherited from `object`**:
`__class__`


|  |  |
| --- | --- |
| Method Details | [hide private] |

|  |  |
| --- | --- |
| \_\_del\_\_(self)  *(Destructor)* | source code |

Overrides: baseclasses.Generator.\_\_del\_\_

|  |  |
| --- | --- |
| \_\_getstate\_\_(self) | source code |

Overrides: baseclasses.Generator.\_\_getstate\_\_

|  |  |
| --- | --- |
| \_\_init\_\_(self, kw)  *(Constructor)* | source code |

x.\_\_init\_\_(...) initializes x; see x.\_\_class\_\_.\_\_doc\_\_ for
signature

Overrides: object.\_\_init\_\_: *(inherited documentation)*

|  |  |
| --- | --- |
| \_\_setstate\_\_(self, state) | source code |

Overrides: baseclasses.Generator.\_\_setstate\_\_

|  |  |
| --- | --- |
| cleanupMemory(self) | source code |

Clean up memory usage from past runs of a solver that is interfaced
through a dynamic link library. This will prevent the 'continue'
integration option from being accessible and will delete other data about
the last integration run.

|  |  |
| --- | --- |
| haveJacobian(self) | source code |

Report whether ODE system has an explicit user-specified Jacobian
associated with it.

Overrides: baseclasses.Generator.haveJacobian

|  |  |
| --- | --- |
| haveJacobian\_pars(self) | source code |

Report whether ODE system has an explicit user-specified Jacobian with
respect to pars associated with it.

Overrides: baseclasses.Generator.haveJacobian\_pars

|  |  |
| --- | --- |
| set(self, \*\*kw) | source code |

Set ODE system parameters

Overrides: baseclasses.Generator.set

  


|  |  |
| --- | --- |
| Class Variable Details | [hide private] |

### \_optionalKeys

Value:
:   ```
    ['globalt0',
     'checklevel',
     'model',
     'abseps',
     'eventPars',
     'FScompatibleNames',
     'FScompatibleNamesInv',
     'tdomain',
    ...
    ```

### \_validKeys

Value:
:   ```
    ['globalt0',
     'xdomain',
     'tdata',
     'tdomain',
     'checklevel',
     'ics',
     'pars',
     'algparams',
    ...
    ```

  


| Home | Trees | Indices | Help | PyDSTool |
| --- | --- | --- | --- | --- |

|  |  |
| --- | --- |
| Generated by Epydoc 3.0.1 on Fri May 4 15:24:06 2012 | http://epydoc.sourceforge.net |
